# Role of Signal Regulatory Protein α in Arsenic Trioxide-induced Promyelocytic Leukemia Cell Apoptosis

**DOI:** 10.1038/srep23710

**Published:** 2016-03-24

**Authors:** Chaoyun Pan, Dihan Zhu, Jianjiang Zhuo, Limin Li, Dong Wang, Chen-Yu Zhang, Yuan Liu, Ke Zen

**Affiliations:** 1 State Key Laboratory of Pharmaceutical Biotechnology, Nanjing Advanced Institute for Life Sciences, School of Life Sciences, Nanjing University, Nanjing, Jiangsu 210093, China; 2 Jiangsu Engineering Research Center for MicroRNA Biology and Biotechnology, 22 Hankou Road, Nanjing, Jiangsu 210093, China; 3 Department of Biology, Georgia State University, Atlanta, GA 30303.

## Abstract

Signal regulatory protein α (SIRPα) has been shown to operate as a negative regulator in cancer cell survival. The mechanism underneath such function, however, remains poorly defined. In the present study, we demonstrate that overexpression of SIRPα in acute promyelocytic leukemia (APL) cells results in apoptosis possibly via inhibiting the β-catenin signaling pathway and upregulating Foxo3a. Pharmacological activation of β-catenin signal pathway attenuates apoptosis caused by SIRPα. Interestingly, we also find that the pro-apoptotic effect of SIRPα plays an important role in arsenic trioxide (ATO)-induced apoptosis in APL cells. ATO treatment induces the SIRPα protein expression in APL cells and abrogation of SIRPα induction by lentivirus-mediated SIRPα shRNA significantly reduces the ATO-induced apoptosis. Mechanistic study further shows that induction of SIRPα protein in APL cells by ATO is mediated through suppression of c-Myc, resulting in reduction of three SIRPα-targeting microRNAs: miR-17, miR-20a and miR-106a. In summary, our results demonstrate that SIRPα inhibits tumor cell survival and significantly contributes to ATO-induced APL cell apoptosis.

SIRPα (also designated as CD172a, p84, SHPS-1) is a receptor-like membrane protein mainly present on mature myeloid leukocytes including neutrophils, monocytes, and macrophage^1^,^2^. As an immunoglobulin superfamily member, SIRPα consists of three extracellular IgV-like loops and a cytoplasmic region with two immunoreceptor tyrosine-based inhibitory motifs (ITIMs). Previous studies have demonstrated that ligation of SIRPα by its ligand CD47, a ubiquitous cell membrane protein, leads to phosphorylation of its ITIMs, which in turn, recruits SH2 domain–containing protein tyrosine phosphatases SHP-1 or SHP-2 to initiate downstream inhibitory signal^3^. It has been shown that, through recruiting and activating SHP-1, SIRPα dephosphorylates Akt and GSK3β, leading to the destabilization of β-catenin and the inactivation of Wnt/β-catenin pathway. For example, Maekawa *et al*. reported that β-catenin was significantly induced by suppressing SIRPα level in K562 cells^4^. Qin *et al*. also reported that SIRPα significantly decreased the expression of β-catenin in hepatocellular carcinoma cell^5^. As canonical Wnt/β-catenin signaling plays an important role in modulating proliferation and survival of tumor cells, particularly the leukemia cells^6^,^7^,^8^,^9^, we speculate that SIRPα may promote acute promyelocytic leukemia (APL) cell apoptosis and suppress APL cell survival via inhibiting Wnt/β-catenin signal pathway.

Arsenic trioxide (As_2_O_3_; ATO), a compound therapeutically used in Chinese traditional medicine, is effective in the treatment of patients with APL^10^,^11^,^12^,^13^. Most APL cases are caused by the chromosomal translocation, resulting in the rearrangement of the promyelocytic leukemia (*PML*) gene and retinoic acid receptor (*RARα*) gene and the production of PML-RARα fusion protein^14^. PML-RARα fusion protein is crucial for the pathogenesis of APL because it operates as a transcriptional silencer in the retinoic acid signaling pathway to block cell differentiation. While displaying a similar role of all-trans-retinoic acid (ATRA) in promoting the degradation of PML-RARα fusion protein^15^, ATO is also a potent inducer of APL cell apoptosis^16^,^17^. It has been reported that ATO induces cell apoptosis through producing reactive oxygen species to activate Chk2/p53 and p38 MAPK/p53 apoptotic pathways^18^ and inhibiting hTERT expression^19^. However, the mechanism underlying the ATO-induced APL cell apoptosis remains incompletely understood.

In the present study, we investigated the general pro-apoptotic effect of SIRPα on tumor cells, and as an extension, we studied the role of SIRPα in the ATO-induced apoptosis of APL cells. We found that expression of SIRPα resulted in apoptosis both of APL HL-60 cells and hepatocellular carcinoma Huh7 cells possibly by suppressing β-catenin signal pathway and upregulating Foxo3a. SIRPα-induced apoptosis could be reversed by pharmacological activation of β-catenin signal pathway. We also observed that ATO treatment induced SIRPα expression in APL cells in a time-dependent manner and abrogation of SIRPα induction prevented APL cell apoptosis and left the β-catenin signaling pathway unperturbed by the ATO treatment. The present study also further explored the miRNA-based mechanism that governs the induction of SIRPα by ATO in APL cells.

## Results

### SIRPα promoted the apoptosis and suppressed the proliferation of APL and hepatocellular carcinoma cells

**Figure 1:**
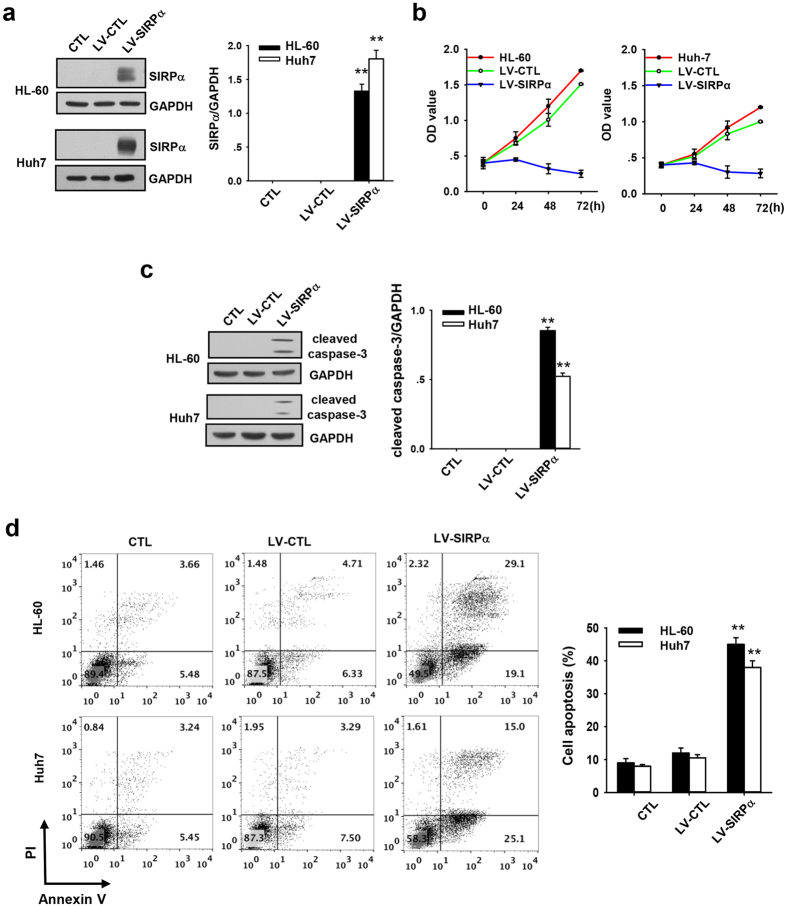
Forced SIRPα expression inhibited proliferation and caused apoptosis in APL and hepatocellular carcinoma cell line. (**a**) Western blotting of SIRPα level in HL-60 and Huh7 cells 48 h post-infection with SIRPα overexpression lentivirus, LV-SIRPα and control lentivirus, LV-CTL. (**b**) Growth curves of HL-60 and Huh7 cells infected with LV-SIRPα or LV-CTL. MTT assays were used to measure cell proliferation on days 1, 2, 3 post-infection. Absorbance was measured at 492 nm. (**c**) Western blotting of cleaved caspase-3 level in HL-60 and Huh7 cells infected with LV-SIRPα or LV-CTL on the third day post infection. (**d**) Flow cytometry analysis of apoptosis of HL-60 and Huh7 cells infected with LV-SIRPα or LV-CTL on the third day post infection with annexin V-PI staining: representative flow cytometer results (left panel) and quantitative analysis of apoptosis (right panel). Values were shown as the mean ± SEM (n = 3). *P < 0.05. **P < 0.01.

Previous studies by us and others showed that APL HL-60 cells and hepatocellular carcinoma Huh7 cells lack SIRPα protein expression. To elucidate the effect of SIRPα, HL-60 and Huh7 cells were infected with either SIRPα-expressing lentivirus (LV-SIRPα) or control lentivirus (LV-CTL) and subjected to cell proliferation and apoptosis assay. The infection efficiency was approximately 90% with MOI 5 (supplementary Figure S1). As shown in Fig. 1a, HL-60 and Huh7 cells infected with LV-SIRPα both expressed significant amount of SIRPα protein at 48 h post-infection. MTT assay on day 3 demonstrated that the proliferation of cells infected with LV-SIRPα was significantly inhibited (Fig. 1b). Furthermore, we monitored the cell apoptosis on day 3 post-infection. The level of cleaved caspase-3, an activated form of caspase-3 was examined by western blotting using anti-caspase-3 antibody, while annexin V on cell surface was evaluated using annexin V-PI kit and flow cytometry. As shown in Fig. 1c, expression of SIRPα in HL-60 and Huh7 cells resulted in a drastic increase in the level of activated capase-3, suggesting that the cells infected with LV-SIRPα underwent apoptosis. Consistent with this finding, HL-60 cells infected with LV-SIRPα displayed a significant increase in the percentage of annexin V-positive cells compared to cells infected with LV-CTL (Fig. 1d). Taken together, our results show that SIRPα expression inhibits the proliferation and promotes the apoptosis of tumor cells.

### Downregulation of β-catenin by SIRPα contributes to tumor cell apoptosis

**Figure 2:**
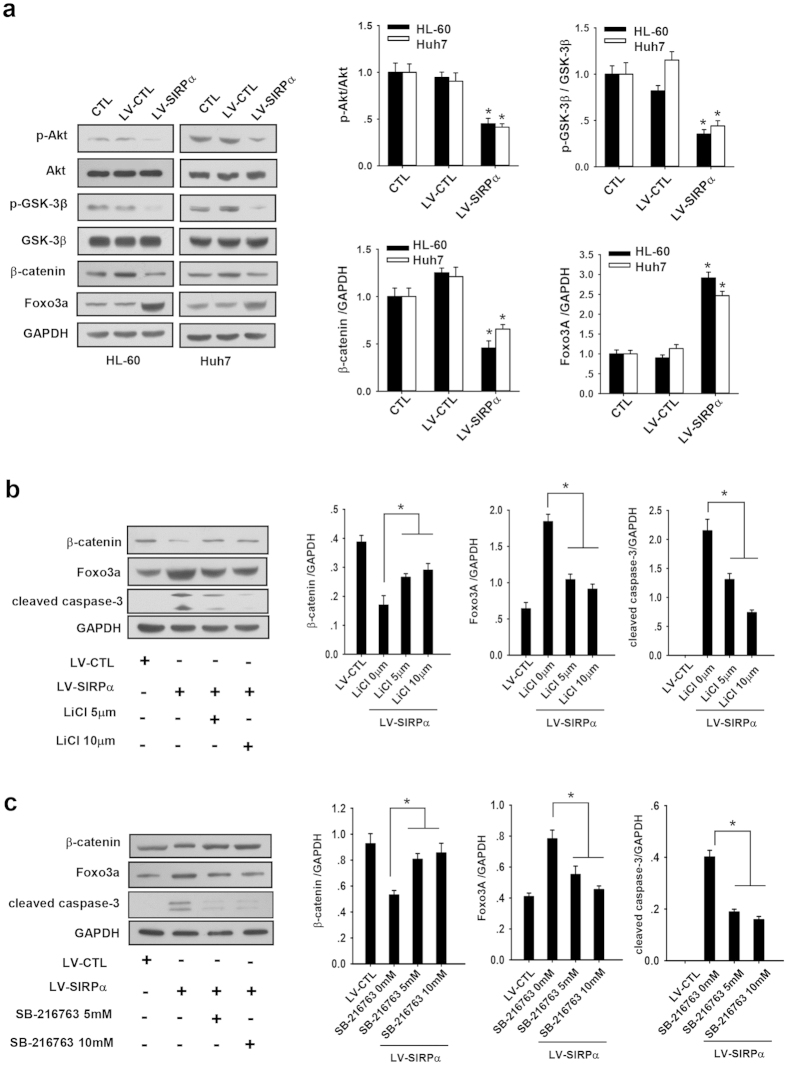
SIRPα contributed to Foxo3a expression and apoptosis possibly by inhibiting β-catenin. (**a**) Western blotting of p-Akt, Akt, p-GSK-3β, GSK-3β, β-catenin, Foxo3a and GAPDH in HL-60 and Huh7 cells infected with LV-SIRPα or LV-CTL on the third day post infection: representative Western blotting (left panels) and quantitative analysis (right panels). (**b**) Western blotting of β-catenin, Foxo3a, cleaved caspase-3 and GAPDH in LV-SIRPα-infected HL-60 cells co-incubated with 0, 5 and 10 μM LiCl or 0, 5, or 10 mM SB-216763. 48 hours post-infection of lentivirus, cells were treated with LiCl, or SB-216763 for 4 h and then lysed for Western blotting analysis: representative Western blot (left panels) and quantitative analysis (right panels). Values were shown as the mean ± SEM (n = 3). *P < 0.05. **P < 0.01.

The involvement of Wnt/β-catenin signal pathway in cell survival and various malignancies has been widely shown^20^,^21^. To explore the mechanism underlying the inhibitory effect of SIRPα on cell survival, we examined whether SIRPα expression could possibly suppress β-catenin expression. For this experiment, we infected HL-60 cells and Huh7 cells with either LV-SIRPα or LV-CTL, and harvested the cells 3 days post-infection for western blotting analysis. As shown in the Fig. 2a, the level of β-catenin was significantly reduced in the HL-60 cells infected with LV-SIRPα but not LV-CTL. As the Wnt/β-catenin signaling pathway can be regulated by Akt/GSK-3β signaling^22^, we further tested whether SIRPα expression disrupted the Akt/GSK3β pathway by measuring the levels of p-Akt, Akt, p-GSK3β and GSK3β in the HL-60 cells infected with LV-SIRPα. As shown in Fig. 2a, levels of p-Akt and p-GSK3β were significantly reduced in HL-60 cells infected with LV-SIRPα, suggesting that SIRPα expression decreased the level of p-Akt and p-GSK3β, leading to activation of GSK3β and the degradation of β-catenin. Since Wnt/β-catenin signaling has been shown to antagonize Foxo3a-mediated apoptosis^23^,^24^, we also assessed whether SIRPα expression could lead to the increased expression of Foxo3a. As shown in Fig. 2a, expression of SIRPα alone significantly promoted the Foxo3a expression in HL-60 cells. Similar results were obtained in the hepatocellular carcinoma Huh7 cells (Fig. 2a). Taken together, these data suggest that SIRPα expression possibly suppress Wnt/β-catenin signaling and promote Foxo3a expression in tumor cells.

To confirm that SIRPα expression promotes the expression of Foxo3a and cell apoptosis through suppressing the level of β-catenin, we tested whether the lithium chloride (LiCl) and SB-216763, two reagents widely used to repress the activity of GSK3β and thus inhibit GSK3β-mediated degradation of β-catenin^23^, can abolish the inhibitory effect of SIRPα expression on β-catenin. As shown in the Fig. 2b, expression of SIRPα alone in the HL-60 cells suppressed β-catenin and promoted cell apoptosis as evidenced by enhanced Foxo3a expression and increased caspase-3 cleavage level. In contrast, treatment with LiCl or SB-216763 in LV-SIRPα-infected cells strongly rescued the β-catenin expression, hampered the increase of Foxo3a expression and blunted the Foxo3A-induced caspase 3 cleavage, suggesting the role of SIRPα in promoting cell apoptosis via reducing β-catenin and enhancing Foxo3a expression.

### Involvement of SIRPα in ATO-induced APL cell apoptosis

**Figure 3:**
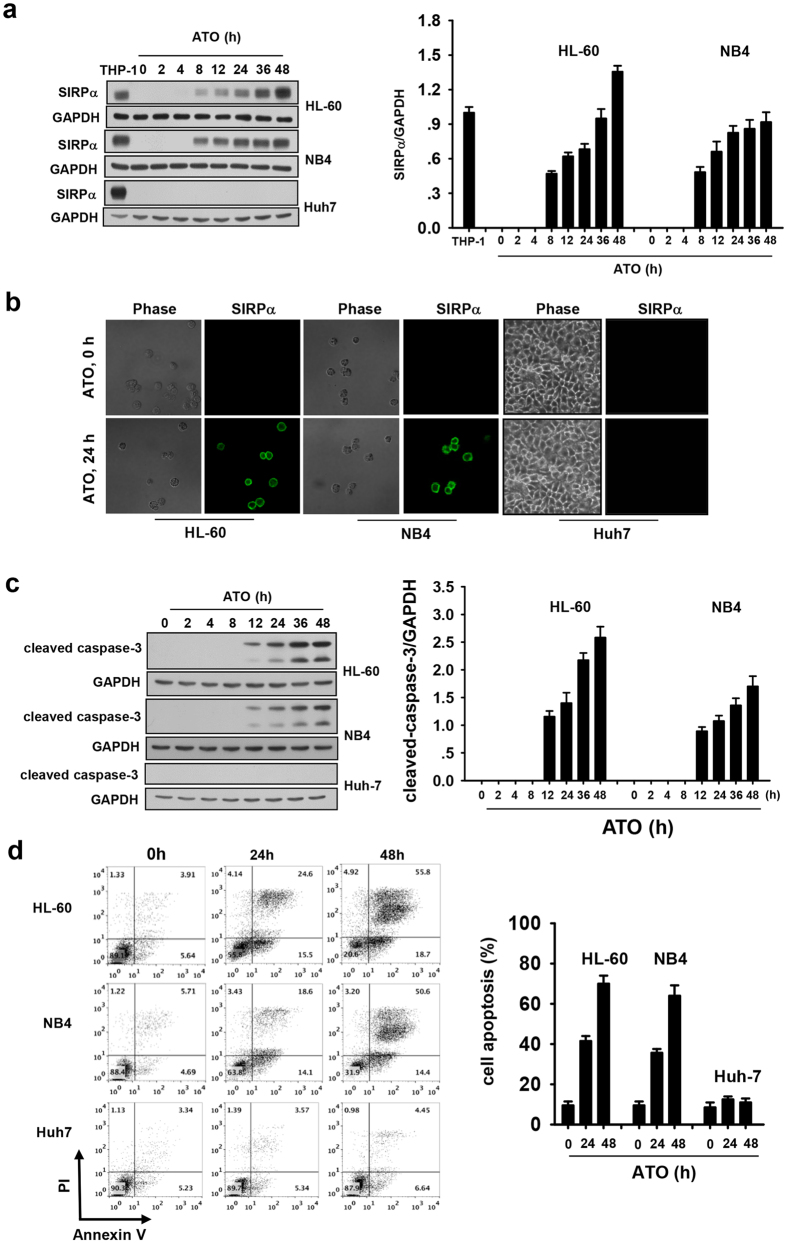
ATO induced expression of SIRPα protein and apoptosis in APL cell lines but not in hepatocellular carcinoma cell line. (**a**) Western blotting of SIRPα level in HL-60, NB4 and Huh7 cells treated with ATO for indicated time, the THP-1 whole cell lysate was used as a positive control: representative Western blotting (left panel) and quantitative analysis of SIRPα level (right panel). (**b**) Immunofluorescence analysis of SIRPα protein induced in HL-60, NB4 and Huh7 cells with ATO treatment for 24 h. (**c**) Cleaved caspase-3 level in HL-60, NB4 and Huh7 cells treated with ATO at indicated time: representative Western blot (left panel) and quantitative analysis (right panel). (**d**) Flow cytometry analysis of ATO-treated HL-60, NB4 and Huh7 cells for indicated time with annexin V-PI staining: representative flow cytometer data (left panel) and quantitative analysis of apoptosis (right panel). The percentage of annexin V positive cells was calculated. Values were shown as the mean ± SEM (n = 3). *P < 0.05. **P < 0.01.

To prove the pro-apoptotic effect of SIRPα on APL cells, we investigated the role of SIRPα in ATO-induced apoptosis of APL cells. We previously reported that APL cell lines, HL-60 and NB4, express no or little SIRPα protein despite harboring a significant amount of SIRPα mRNA^25^. However, to our surprise, ATO can induce a *de novo* expression of SIRPα protein in both HL-60 and NB4 cells. As shown in the Fig. 3a, treatment of HL-60 and NB4 cells with ATO triggered a significant induction of SIRPα in a time-dependent manner. SIRPα protein was detectable within 8 h and reached peak level after 48 h of ATO treatment. Immunofluorescence analysis further showed that SIRPα protein induced by ATO treatment was correctly transported to the cell surface (Fig. 3b). Moreover, the induction of SIRPα in HL-60 and NB4 cells by ATO was positively correlated with the ATO-induced apoptosis. As shown in the Fig. 3c,d, ATO treatment led to an increase in cleaved capase-3 level in a time-dependent manner. Treatment of APL cells with ATO was also found to induce a strong increase in the percentage of Annexin V-positive cells. These results are in agreement with previous reports that APL cells are susceptible to the apoptosis induced by ATO treatment^26^. Interestingly, we found that, unlike APL cells, hepatocellular carcinoma Huh7 cells were not sensitive to ATO treatment and displayed no enhanced apoptosis induced by the same concentration of ATO within 48 h (Fig. 3c,d). Accordingly, no induction of SIRPα in Huh7 cells was observed in the process of ATO treatment (Fig. 3a,b). Taken together, these results suggest that ATO-induced apoptosis might be mediated by SIRPα expression.

**Figure 4:**
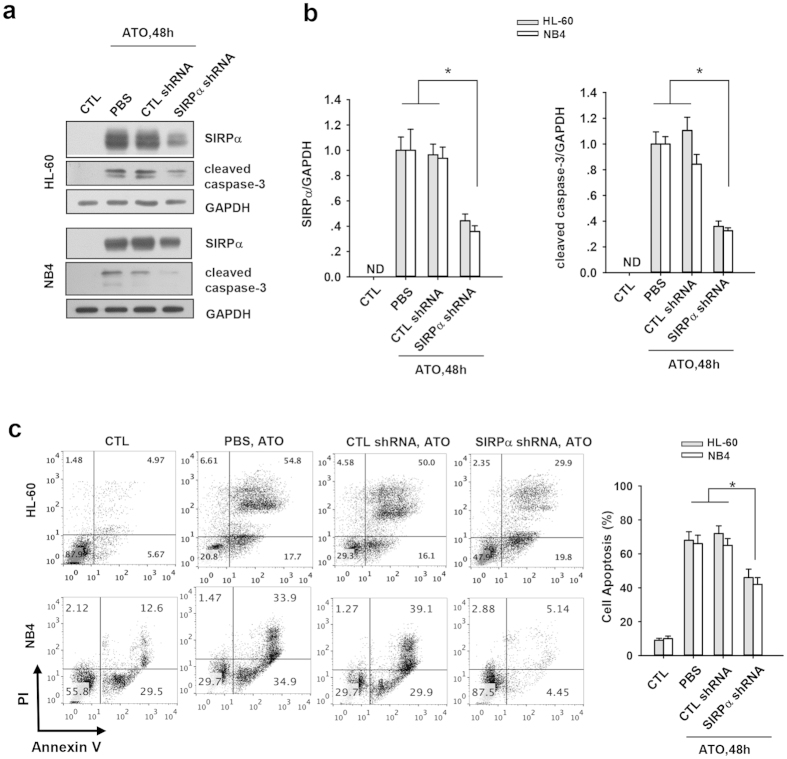
Block of SIRPα induction attenuated ATO-induced apoptosis of APL cell lines. SIRPα and cleaved caspase-3 protein level in SIRPα shRNA lentivirus-infected HL-60 or NB4 cells treated with ATO for indicated time: representative Western blots (**a**) and quantitative analysis of Western blot (**b**). Cells treated without lentivirus (PBS) or the cells infected with CTL shRNA lentivirus were used as controls. (**c**) Flow cytometry analysis of annexin V-PI staining in SIRPα shRNA lentivirus-infected HL-60 or NB4 cells in the presence of ATO for indicated time. Left panel, representative flow cytometer data. Right panel, quantitative analysis of apoptosis. Values were shown as the mean ± SEM (n = 3). *P < 0.05. **P < 0.01.

We next determined whether the induction of SIRPα by ATO treatment directly contributed to the cell apoptosis. In these experiments, we used a lentivirus-mediated SIRPα siRNA (SIRPα shRNA) to specifically abolish the induction of SIRPα protein in both HL-60 and NB4 cells by ATO. As shown in the Fig. 4a,b, SIRPα shRNA successfully decreased the induction of SIRPα protein in both HL-60 and NB4 cells by ATO treatment. More importantly, abrogation of ATO-induced SIRPα expression by SIRPα shRNA also blocked the ATO-mediated cell apoptosis, as shown by decreased caspase-3 cleavage (Fig. 4b,d). In agreement with this, Annexin V staining also showed that the percentage of Annexin V-positive cells in ATO-treated HL-60 and NB4 cells were decreased after SIRPα was knocked down with SIRPα shRNA (Fig. 4e). These results collectively suggest that SIRPα possibly mediates ATO-induced apoptosis of APL cells.

**Figure 5:**
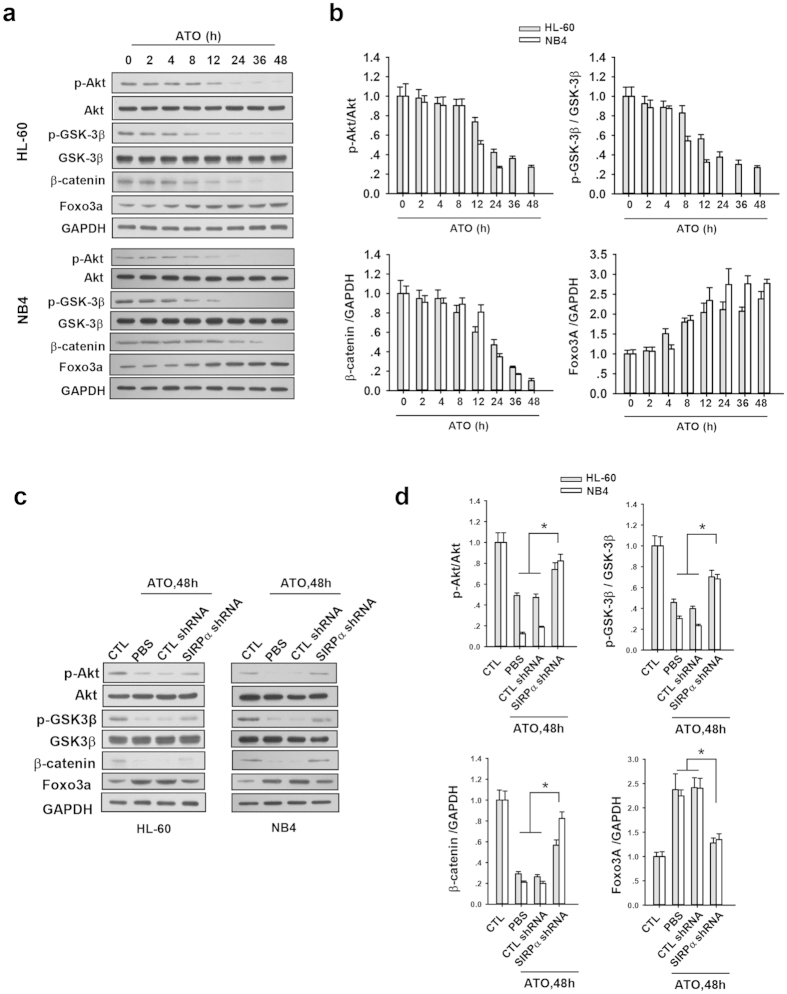
Induction of SIRPα contributed to the downregulation of β-catenin and upregulation of Foxo3a in APL cell lines treated with ATO. (**a,b**) Western blotting of p-Akt, Akt, p-GSK-3β, GSK-3β, β-catenin, Foxo3a and GAPDH in HL-60 or NB4 cells treated with ATO for indicated time: representative Western blotting (**a**) and quantitative analysis of protein level (**b**), the protein level of GAPDH was used as an internal control, the p-Akt (Ser473) and p-GSK-3β (Ser9) were normalized to the total Akt and GSK-3β, respectively. (**c,d**) Western blotting of p-Akt, Akt, p-GSK-3β, GSK-3β, β-catenin, Foxo3a and GAPDH in SIRPα shRNA lentivirus infected HL-60 or NB4 cells after the treatment of ATO for 48 h: representative Western blot (**c**) and quantitative analysis (**d**). Values were shown as the mean ± SEM (n = 3). *P < 0.05. **P < 0.01.

To test whether the contribution of SIRPα on ATO-induced apoptosis is possibly through inhibiting β-catenin signal pathway, we studied the effect of SIRPα on β-catenin levels in both HL-60 and NB4 cells that were treated with ATO. As expected, treatment of HL-60 and NB4 cells with ATO alone resulted in a drastic suppression of β-catenin, as assessed by western blotting (Fig. 5a,b). Consistent with the suppression of β-catenin, the phosphorylation of Akt and GSK-3β was reduced but the expression of Foxo3a significantly increased by ATO treatment (Fig. 5a,b). To confirm that SIRPα induction is required for the suppression of β-catenin and upregulation of Foxo3a in HL-60 and NB4 cells by ATO, we knocked down SIRPα in both HL-60 and NB4 cells using SIRPα shRNA lentivirus and then evaluated the expression of β-catenin, as well as the expression of Foxo3a and the phosphorylation status of Akt and GSK-3β. As shown in the Fig. 5c,d, knockdown of SIRPα in HL-60 or NB4 cells treated with ATO significantly enhanced the phosphorylation of Akt and GSK-3β, leading to increase of β-catenin level but decrease of Foxo3a expression.

### Induction of SIRPα by ATO is through suppression of miR-17, miR-20a and miR-106a

**Figure 6:**
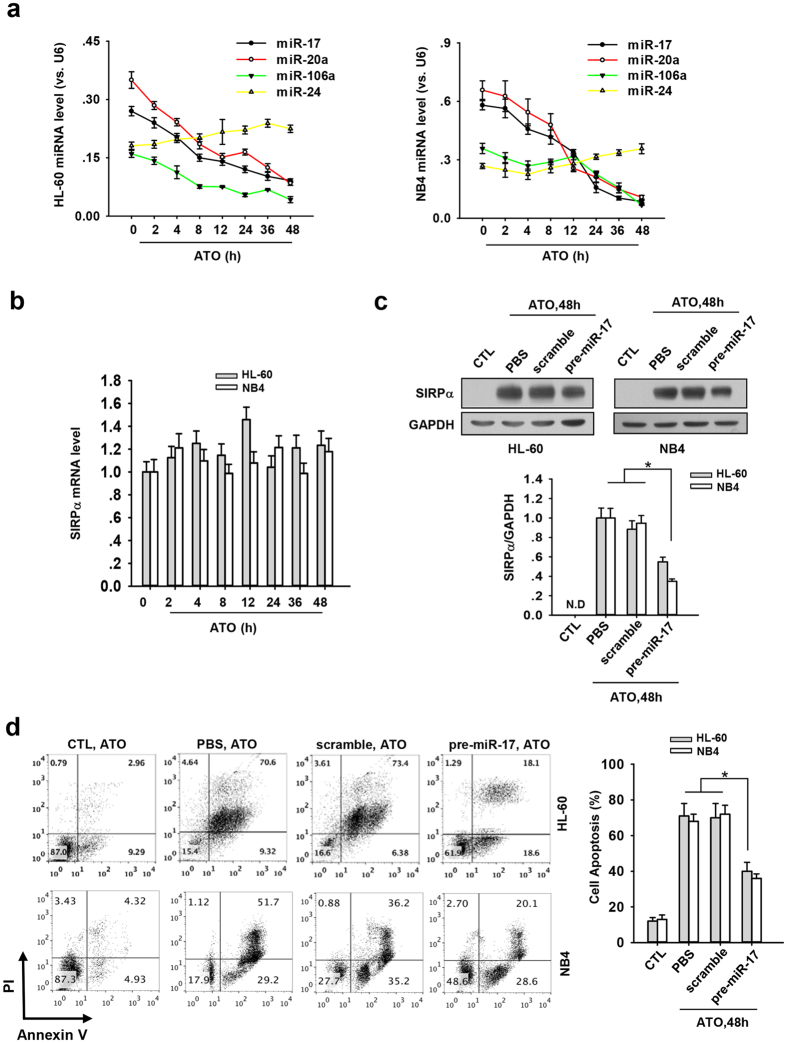
ATO induced expression of SIRPα through the suppression of miR-17, miR-20a, and miR-106a. (**a**) The level of SIRPα-regulating miRNAs, miR-17, miR-20a, miR-106a in HL-60 or NB4 cells treated with ATO at indicated time. The level of all miRNAs was normalized to that of U6. (**b**) The relative mRNA level of SIRPα in HL-60 or NB4 cells treated with ATO for indicated time. Total RNA was extracted from the cells and analyzed with RT-qPCR. The mRNA level of GAPDH was used as an internal control. (**c**) Western blotting of SIRPα protein level in the HL-60 or NB4 cells treated with ATO for indicated time. Before ATO treatment, the cells were transfected with pre-miR-17. The mock-transfected cells (PBS) or cells transfected with scrambles oligonucleotide were used as a control: representative Western blot (upper panel) and quantitative analysis (lower panel). (**d**) Flow cytometry analysis of apoptosis of pre-miR-17-transfected HL-60 or NB4 cells after the treatment of ATO for 48 h: representative flow cytometer data (left panel) and quantitative analysis of apoptosis (right panel). The percentage of annexin V-positive cells was calculated. Values were shown as the mean ± SEM (n = 3). *P < 0.05. **P < 0.01.

As our previous study showed that SIRPα was post-transcriptionally regulated by a cluster of miRNAs, viz., miR-17, miR-20a, and miR-106a^25^, we next determined whether these miRNAs were involved in modulating APL cell SIRPα protein levels in response to ATO. To address this, we assayed the level of these miRNAs in both HL-60 and NB4 cells treated with ATO. The expression of miR-17, miR-20a and miR-106a was decreased in a time-dependent manner (Fig. 6a), while mRNA level of SIRPα was largely not affected by ATO treatment (Fig. 6b). As a control, the level of miR-24, a randomly selected miRNA, was largely unchanged (Fig. 6a). To confirm the role of miR-17, miR-20a and miR-106a in regulating SRIPα protein expression in APL cells during ATO-induced apoptosis, we transfected both HL-60 and NB4 cells with pre-miR-17 48 hours prior to the ATO treatment. As shown in Fig. 6c, HL-60 or NB4 cells transfected with pre-miR-17 expressed significantly less SIRPα compared to non-transfected cells or cells transfected with scramble oligonucleotide. Accordingly, pre-miR-17-transfected HL-60 or NB4 cells displayed a significantly delayed and attenuated apoptosis compared to the non-transfected cells or cells transfected with scramble oligonucleotide under the same treatment with ATO (Fig. 6d).

**Figure 7:**
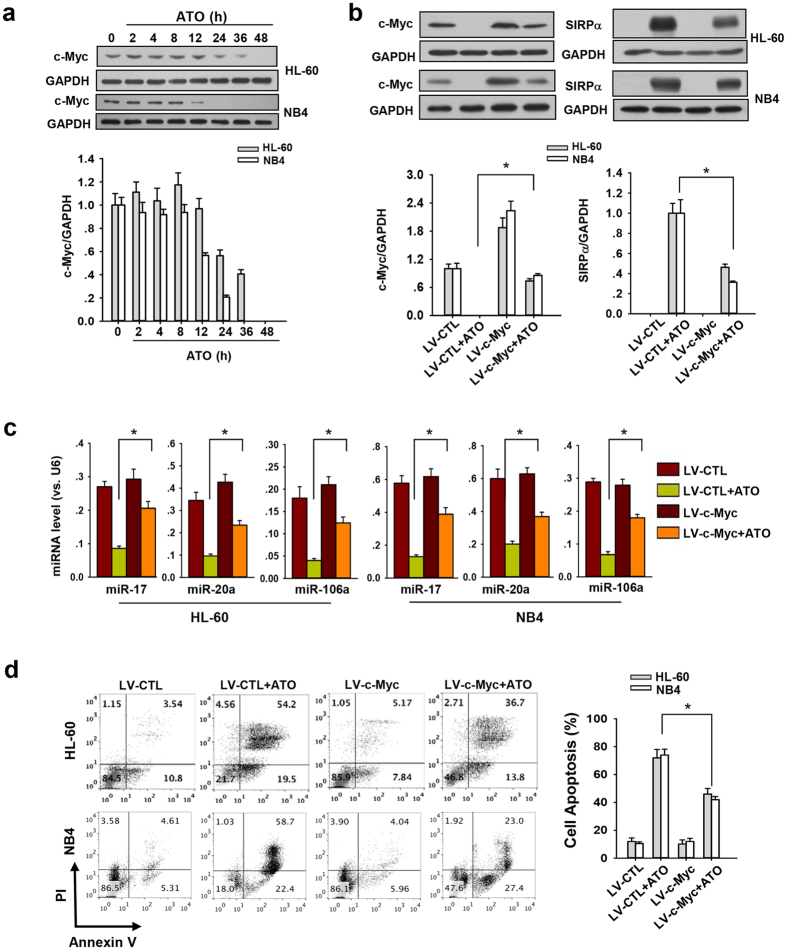
ATO suppressed miR-17, miR20a, and miR-106a through the inhibition of c-Myc expression. (**a**) Western blotting analysis of c-Myc level in HL-60 or NB4 cells treated with ATO for indicated time: representative Western blot (upper panel) and quantitative analysis (lower panel). (**b**) Western blotting of c-Myc and SIRPα in c-Myc overexpression lentivirus (LV-c-Myc) infected HL-60 or NB4 cells after treated with ATO for 48 hours. The lentivirus (LV-CTL) was used as a control: representative Western blotting (upper panel) and quantitative analysis (lower panel). (**c**) The level of SIRPα-regulating miRNAs, miR-17, miR-20a, miR-106a in LV-c-Myc infected HL-60 or NB4 cells after treated with ATO for 48 h, LV-CTL was used as a control. (**d**) Flow cytometry analysis of apoptosis of LV-c-Myc infected HL-60 or NB4 cells after the treatment of ATO for 48 h: representative flow cytometer results (left panel) and quantitative analysis of apoptosis (right panel). The percentage of annexin V-positive cells was calculated. Values were shown as the mean ± SEM (n = 3). *P < 0.05. **P < 0.01.

Our previous report also showed that c-Myc can promote the expression of the miR-17~92 cluster in promyelocytic cells^25^. To test whether ATO suppresses the expression of miR-17, miR20a and miR-106a in promyelocytic cells by reducing c-Myc level, we determined the effect of ATO treatment on c-Myc level, as well as miR-17, miR-20a, and miR-106a expression, in both HL-60 and NB4 cells. As shown in Fig. 7a, c-Myc levels were strongly decreased in HL-60 cells treated with ATO in a time-dependent manner, suggesting a negative regulatory role of c-Myc in SIRPα protein expression in response to ATO treatment. Furthermore, when ATO-induced reduction of c-Myc in HL-60 or NB4 cells was reversed by transfection with lentivirus-mediated c-Myc expression vector (LV-c-Myc), the induction of SIRPα protein by ATO treatment was largely abolished (Fig. 7b). The downregulation of miR-17, miR-20a and miR-106a in ATO-treated HL-60 or NB4 cells was also reversed by overexpression of c-Myc (Fig. 7c). As shown in Fig. 7d, restoration of c-Myc level in ATO-treated HL-60 or NB4 cells significantly reduced cell apoptosis. These results suggest that ATO downregulates miR-17, miR-20a, and miR-106a possibly by suppressing c-Myc subsequent to which SIRPα expression is induced in APL cells.

## Discussion

As a critical signal transduction protein, SIRPα has been shown to be involved in regulating many aspects of cellular responses, particularly the inflammatory responses of leukocytes, including activation, chemotaxis and phagocytosis^27^,^28^,^29^,^30^,^31^,^32^. A correlation between SIRPα and cell growth and survival was reported recently by Yan *et al*.^33^ who showed that ectopic expression of SIRPα in hepatocellular carcinoma suppressed cell growth. In the present study, we demonstrated SIRPα’s role as a pro-apoptotic molecule that mediates ATO-induced apoptosis of APL cells.

Several pieces of evidence support this previously unrecognized role of SIRPα in ATO-induced apoptosis. First, SIRPα expression was induced by ATO treatment in APL cells in a time-dependent manner. Immunofluorescence staining showed that most of the SIRPα protein was on the cell surface, where SIRPα could bind to its ligand and initiate downstream signaling. More importantly, specific knockdown of ATO-induced SIRPα expression via lentivirus-mediated SIRPα shRNA largely blocked ATO-induced APL cell apoptosis. Second, although ATO treatment can effectively induce apoptosis of APL cells, it shows limited effect on other malignancies particularly solid tumors^16^. Here we also found that hepatocellular carcinoma Huh7 cells, unlike APL cells, were insensitive to ATO treatment. Serving a negative control, Huh7 cells displayed no induction of SIRPα by ATO.

Overexpression of SIRPα alone in APL cells (HL-60 and NB4) or hepatocellular carcinoma Huh7 cells significantly increased cell apoptosis, strongly arguing that SIRPα is a general pro-apoptotic molecule functioning in various tumor cells. As SIRPα can induce cell apoptosis, we speculate that induction of SIRPα in APL cells is not just sensitizing the cells to ATO treatment but is a novel mechanism underneath the ATO-mediated APL cell death. Failure to induce SIRPα in cancer cells such as Huh7 cells yielded the poor effect of ATO treatment on their apoptosis. In an effort to probe into the role of SIRPα in ATO-induced apoptosis in APL cells, we employed a lentivirus system to increase or knock down cellular SIRPα protein level by transfecting cells with lentivirus-mediated SIRPα mRNA or SIRPα shRNA. As shown by our results (Figs 4 and 5), the contribution of SIRPα to the ATO-induced APL cell apoptosis is produced possibly through its role in inhibiting β-catenin signal pathway. Treatment with ATO or overexpression of SIRPα alone decreased the levels of phosphorylated Akt and GSK-3β, leading to suppression of β-catenin level but enhancement of Foxo3a expression. In contrast, knockdown of SIRPα in ATO-treated APL cells significantly rescued the expression of β-catenin, inhibited the increase of Foxo3a and alleviated the cell apoptosis induced by ATO.

Our previous study showed that SIRPα expression was modulated at posttranscriptional level by miR-17, miR-20a, and miR-106a^25^. In the present study, we also observed that the induction of SIRPα was dependent on the suppression of these three miRNAs by ATO treatment. Overexpression of one such miRNA in ATO-treated APL cells significantly abolished the induction of SIRPα by ATO (Fig. 6). The involvement of miR-17, miR-20a and miR-106a in posttranscriptional regulation of SIRPα was also supported by the facts that ATO treatment did not affect SIRPα mRNA level but increased SIRPα protein and reduced c-Myc expression in APL cells (Fig. 7). Given that c-Myc strongly promotes the expression of miR-17, miR-20a, and miR-106a, as indicated by Fig. 6 as well as our previous report^25^, suppression of c-Myc level in APL cells by ATO treatment may play a key role in downregulation of miR-17, miR-20a and miR-106a, and thus upregulation of SIRPα protein translation. Interestingly, since SIRPα protein can inhibit β-catenin signal pathway, which in turn, positively regulates c-Myc transcription^34^,^35^, induction of SIRPα may downregulate c-Myc level, leading to downregulation of miR-17, miR-20a and miR-106a expression but even higher level of SIRPα protein. Therefore, through β-catenin and c-Myc signal pathways, a miRNA-based positive-feedback regulatory loop may be involved in ATO-induced SIRPα induction and cell apoptosis in APL cells.

Given that SIRPα can suppress tumor cell survival and more important, SIRPα is induced by ATO treatment in APL cells, the rapid induction of SIRPα in APL cells may serve as a novel prognostic marker for ATO treatment. In addition, as tumor cells express SIRPα mRNA but no SIRPα protein, induction of SIRPα protein expression in these tumor cells by suppressing the expression of SIRPα-targeting miRNAs also provide a potential anti-tumor strategy.

In conclusion, our results identify SIRPα as an important pro-apoptotic regulator and the induction of SIRPα may play a critical part in mediating ATO-induced apoptosis of APL cells.

## Materials and Methods

### Cell and Reagent

HL-60 and NB4 cells were maintained in RMPI1640 (GIBCO, Carlsbad, CA) supplemented with 10% (v/v) fetal bovine serum (FBS) (GIBCO), 1% penicillin and streptomycin (GIBCO). Huh7 cells were cultured in DMEM (GIBCO) supplemented with 10% (v/v) fetal bovine serum (FBS), 1% penicillin and streptomycin. Antibodies against cleaved caspase-3, Akt, p-Akt (Ser473), GSK-3β, p-GSK-3 (Ser9) and β-catenin were purchased from Cell Signaling Technology (Danvers, MA). Antibodies against GAPDH, SIRPα, and secondary antibodies against mouse or rabbit IgGs were purchased from Santa Cruz Biotechnology (Santa Cruz, CA). Alexa Flour 488 (goat anti-rabbit IgG, green) and annexin V-PI apoptosis kit were purchased from Invitrogen (Carlsbad, CA). Arsenic trioxide (As_2_O_3_; ATO) was purchased from Sigma Aldrich (St Louis, MO). Cells in our experiment were treated with ATO at 5 μM concentration where indicated. Lithium chloride (LiCl) and SB-216763 were purchased from Sigma Aldrich. 48 hours post-infection of lentivirus, cells were treated with 5 μm or 10 μm LiCl, 5 mM or 10 mM SB-216763 for 4 h and then lysed for westert blotting analysis..

### MTT proliferation assay

Cells in each experiment were seeded in 96-well plates at a density of 1.0 × 10^4^ cells/well and infected with SIRPα overexpressing lentivirus or control lentivirus. 48 hours post infection, cell proliferation was quantified in 3 days by MTT assay. In brief, 20μl of MTT (5 mg/ml; Sigma) was added to each well followed by incubation for 4 h at 37°C. The medium was then replaced with 150 μl of dimethylsulphoxide (DMSO) (Sigma). The viability of the cells was assessed by the detection of absorbance at 492 nm using a spectrophotometer. The growth curves were plotted.

### Cell lysis and Immunoblotting

Cell were treated with or without ATO for the indicated time and subsequently lysed in the RIPA lysis buffer (50 mM Tris pH 7.4, 150 mM NaCl, 1% NP-40, 0.5% sodium deoxycholate, 0.1% SDS) supplemented with protease inhibitor cocktail (Amresco, Cleveland, Ohio) and/or phosphatase inhibitor cocktail (Cell Signaling Technology). The whole cell lysis was subjected to SDS-PAGE, transferred onto a PVDF membrane, and then immunoblotted with respective primary antibodies. The bound primary antibody were visualized with specific horseradish peroxidase-conjugated secondary antibodies (Santa Cruz) using an enhanced chemiluminescence (ECL) reagents (Thermo Scientific, Hudson, NH). The bar graphs corresponding to the Western blots were generated through densitometric analysis with Image J software.

### Immunofluorescence Analyses

Cells were harvested after treatment in the presence or absence of ATO for indicated time by centrifugation at 300 × g for 5 minutes at 4 °C and subjected to Immunofluorescence. Briefly, the harvested cells were washed using cold phosphate-buffered saline (PBS) and fixed with 4% paraformaldehyde for 10 minutes at room temperature. Then the cells were washed using PBS and permeabilized with PBS containing 0.2% (v/v) Triton X-100 for 5 minutes at room temperature. After blocking permeabilized cells with bovine serum albumin (BSA) for 1 hour at room temperature, cells were washed using PBS and incubated with SIRPα antibody (1:100) in PBS containing 10 mg/ml BSA overnight at 4 °C. Cells were washed using PBS and then incubated with Alexa Flour 488 (goat anti-rabbit IgG, 1:2000) for 1 h at room temperature. The cells were washed using PBS and then mounted and analyzed with an inverted confocal laser microscope (Nikon).

### RNA isolation and RT-qPCR

Total RNA from cells was extracted using the TRIzol Reagent according to the manufacturer’s protocol (Invitrogen). The relative expression of mRNAs was determined by RT-qPCR using LightCycler FastStart DNA Master SYBR Green I (Roche Diagnostics, Indianapolis, IN) according to the manufacturer’s protocol. The mRNA of GAPDH was used as internal controls. The expression of miRNAs was determined by qRT-PCR using TaqMan miRNA probes (Applied Biosystems, Foster City, CA) according to the manufacturer’s protocol. The levels of miRNAs in cells were normalized to U6 snRNA. All of the reactions were run in triplicate. A comparative threshold cycle (ΔCT) method was used and values were expressed as 2^−ΔΔCT^.

### Flow Cytometric Assays

Cells were treated for the respective time period and harvested by centrifugation at 300 × g for 5 minutes at 4 °C. The annexinV-propidium iodide kit (Invitrogen) was used to stain the cells for the evaluation of apoptosis according to the manufacturer’s protocol.

### Infection with shRNA SIRPα lentivirus, SIRPα-expressing lentivirus and c-Myc-expressing lentivirus

Lentivirus encoding SIRPα, shRNA SIRPα, and c-Myc were generated and confirmed by the GenePharma Company (Shanghai, China). An empty backbone lentivirus was used as a control. Cells were incubated with respective virus at a multiplicity of infection (MOI) of 5 along with 8 μg/ml Polybrene for 48 h before the treatment with indicated dose of ATO. The selection marker was GFP. The infected cells were gated by GFP expression via flow cytometry analysis.

### Statistical analyses

Data derived from at least three independent experiments are expressed as the mean ± SEM. Normal distributed variables were compared using Student’s *t*-test. The reported P value was 2-sided. P < 0.05 was considered to be statistically significant.

## Additional Information

**How to cite this article**: Pan, C. *et al*. Role of Signal Regulatory Protein α in Arsenic Trioxide-induced Promyelocytic Leukemia Cell Apoptosis. *Sci. Rep.* **6**, 23710; doi: 10.1038/srep23710 (2016).

## Supplementary Material

Supplementary Information
