# Supplementary material for: Role of Signal Regulatory Protein α in Arsenic Trioxide-induced Promyelocytic Leukemia Cell Apoptosis
Source: Sci Rep. 2016 Mar 24;6:23710. doi: 10.1038/srep23710 (PMC4806322; doi:10.1038/srep23710)
Supplement: Supplementary Information [file srep23710-s1.pdf]

Role of Signal Regulatory Protein α in Arsenic Trioxide-induced Promyelocytic Leukemia Cell Apoptosis

Chaoyun Pan[1,2+], Dihan Zhu[1,2+], Jianjiang Zhuo[1,2], Limin Li[1,2], Dong Wang[1,2], Chen-Yu Zhang[1,2*], Yuan Liu[3+*], and Ke Zen[1,2,3*]

From [1]State Key Laboratory of Pharmaceutical Biotechnology, Nanjing Advanced Institute for Life Sciences, School of Life Sciences, Nanjing University; [2]Jiangsu Engineering Research Center for MicroRNA Biology and Biotechnology, 22 Hankou Road, Nanjing, Jiangsu 210093, China; [3]Department of Biology, Georgia State University, Atlanta, GA 30303

Running title: *SIRPα mediates APL cell apoptosis*

[+]These authors contributed equally to this work.

*Corresponding authors:

Ke Zen, PhD, Chen-Yu Zhang, MD, PhD, and/or Yuan Liu, MD, PhD,

E-mail: kzen@nju.edu.cn, cyzhang@nju.edu.cn, yliu@gsu.edu

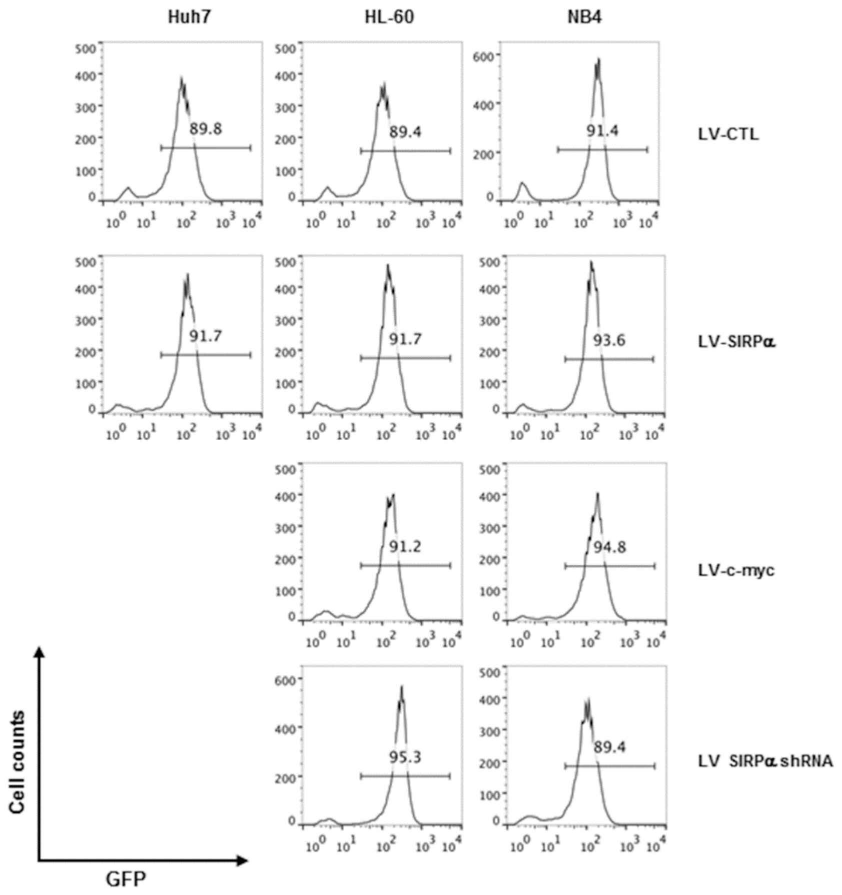

Supplemental Figure S1. Representative results of all the lentivirus infection efficiency. Cells were incubated with respective virus at a multiplicity of infection (MOI) of 5 along with 8 μg/ml Polybrene for 48 h before the treatment with indicated dose of ATO. The selection marker was GFP. The infected cells were gated by GFP expression via flow cytometry analysis.
